# Post-Translational Modifications Modulate Ligand Recognition by the Third PDZ Domain of the MAGUK Protein PSD-95

**DOI:** 10.1371/journal.pone.0090030

**Published:** 2014-02-26

**Authors:** Javier Murciano-Calles, Carles Corbi-Verge, Adela M. Candel, Irene Luque, Jose C. Martinez

**Affiliations:** Department of Physical Chemistry and Institute of Biotechnology, Faculty of Sciences, University of Granada, Granada, Spain; Universitat Pompeu Fabra, Barcelona Research Park of Biomedicine (PRBB), Spain

## Abstract

The relative promiscuity of hub proteins such as postsynaptic density protein-95 (PSD-95) can be achieved by alternative splicing, allosteric regulation, and post-translational modifications, the latter of which is the most efficient method of accelerating cellular responses to environmental changes *in vivo*. Here, a mutational approach was used to determine the impact of phosphorylation and succinimidation post-translational modifications on the binding affinity of the postsynaptic density protein-95/discs large/zonula occludens-1 (PDZ3) domain of PSD-95. Molecular dynamics simulations revealed that the binding affinity of this domain is influenced by an interplay between salt-bridges linking the α3 helix, the β2–β3 loop and the positively charged Lys residues in its high-affinity hexapeptide ligand KKETAV. The α3 helix is an extra structural element that is not present in other PDZ domains, which links PDZ3 with the following SH3 domain in the PSD-95 protein. This regulatory mechanism was confirmed experimentally via thermodynamic and NMR chemical shift perturbation analyses, discarding intra-domain long-range effects. Taken together, the results presented here reveal the molecular basis of the regulatory role of the α3 extra-element and the effects of post-translational modifications of PDZ3 on its binding affinity, both energetically and dynamically.

## Introduction

One of the most ambitious goals of modern biology is to understand protein networks that regulate complex cellular processes. The main distinctive feature of these networks is the presence of hubs and super-hubs that undergo hundreds or thousands of different interactions [1]. Such proteins are able to recognise and distinguish between a variety of targets, are evolutionary conserved, and play central roles in specific processes [2]. Promiscuity of hub proteins can be achieved in a number of different ways; for example, alternative splicing can lead to the generation of a large number of isomers that enable multiple protein-protein interactions [3], [4]. Allosteric regulation and post-translational modifications constitute a way of multiplying functionalities by modulating the affinities and specificities of protein binding sites. In fact, post-translational modifications are the most efficient method to accelerate the cellular response to environmental changes [5].

**Figure 1:**
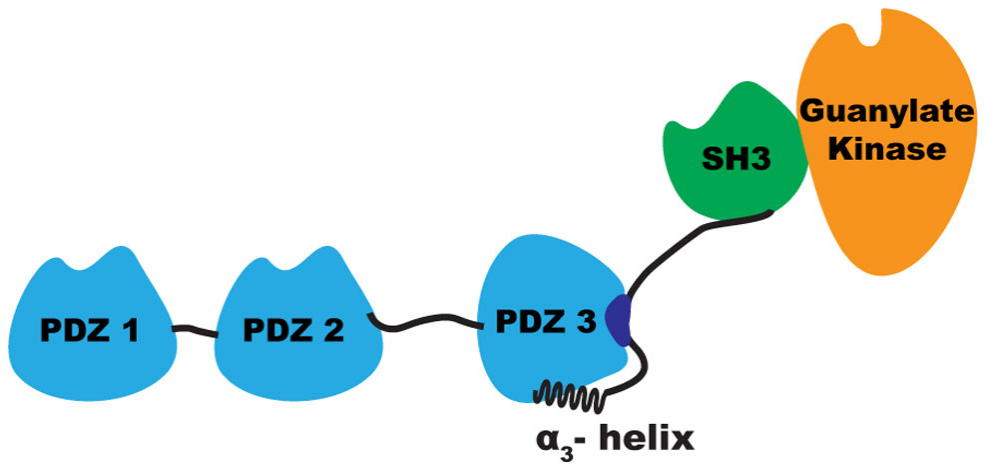
The ‘supertertiary’ structure of PSD-95 protein.

Members of the membrane-associated guanylate kinase (MAGUK) family, including postsynaptic density protein-95 (PSD-95; also known as SAP-90 or DLG-4), are known hub proteins. MAGUKs lack enzymatic activity and are structurally organised into several conformationally-independent modular domains that are interconnected by relatively short amino acid sequences; these domains typically comprise three postsynaptic density protein-95/discs large/zonula occludens-1 (PDZ) domains, one SH3 domain and one kinase domain (Figure 1). Structural modelling of the multi-modular organisation of PSD-95 has revealed that the protein develops a level of functional regulation through high conformational plasticity that is achieved mainly by the inter-domain sequences [6]. The term ‘supertertiary structure’ has been proposed to describe the multiplicity of conformations and states of these multi-modular proteins that might coexist and interchange under equilibrium conditions [7], [8].

**Figure 2:**
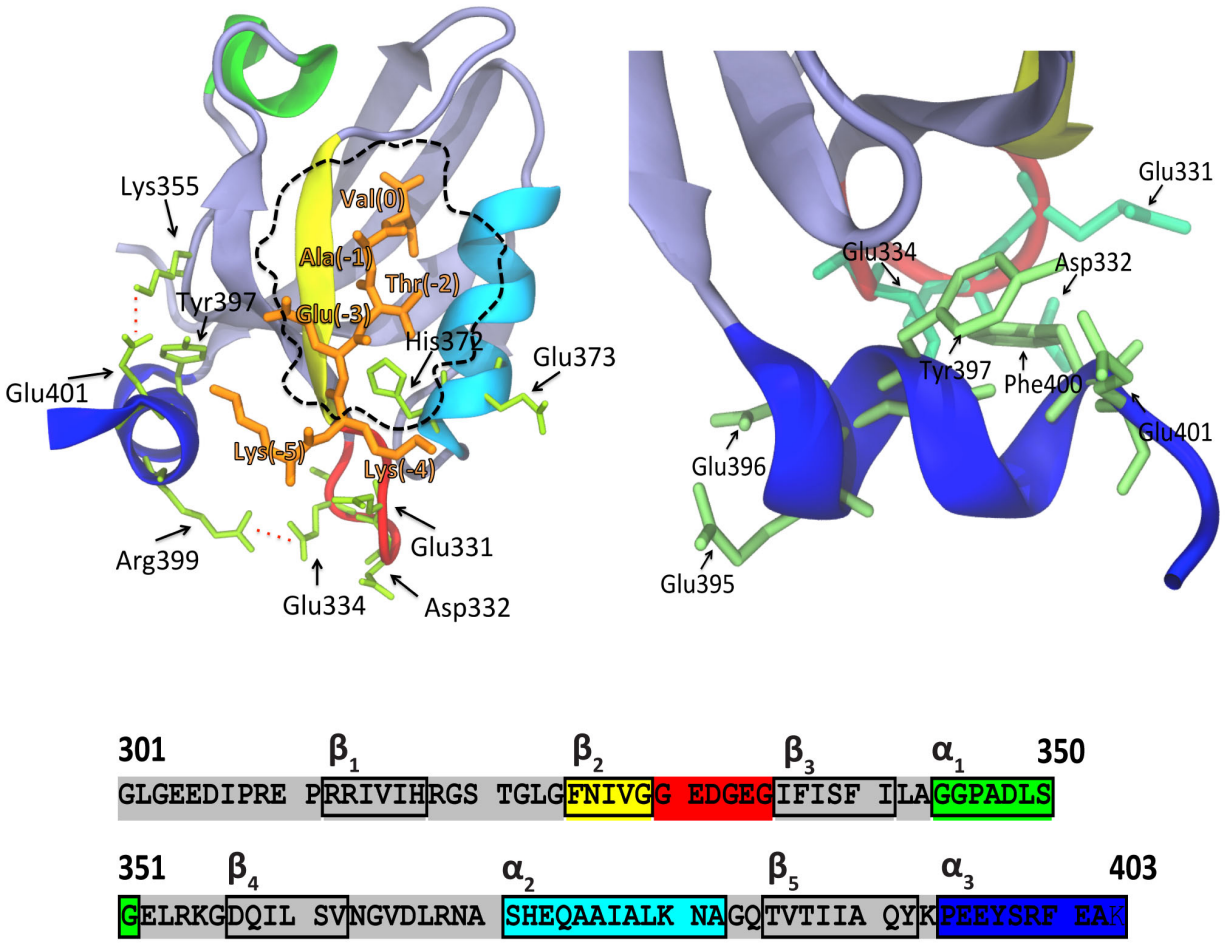
The structure and sequence of the PDZ3 domain of PSD-95. The panel on the upper left shows a structural representation of the PDZ3 domain of PSD-95 in complex with the hexapeptide KKETAV (orange), modelled from the X-ray structure of the PDZ3-CRIPT complex (Protein Data Bank ID: 1BE9). The α1, α2, and α3 helices are shown in green, light blue and blue, respectively. The β2–β3 loop is shown in red and the β2 chain is shown in yellow. The dashed line indicates the binding pocket. The panel on the upper right is a detailed view of the interface of the α3 helix at the C-terminus of PDZ3 showing the spatial arrangement of the Phe, Tyr, Asp, and Glu residues. The lower panel shows the sequence of the PDZ3 domain and its secondary structures. Numbering of the protein residues is relative to their positions in the full-length PSD-95 protein. Numbering of the KKETAV peptide residues is from 0 (C-terminal Val residue) to −5 (N-terminal Lys residue).

An α-helical segment in PSD-95 (Figures 1 and 2; α3 helix), which connects the PDZ3 domain to the subsequent SH3 domain, packs the PDZ3 domain at a different face to that of the binding pocket, thereby enabling allosteric modulation of the protein, as demonstrated by a 21-fold drop in affinity for the C-terminal sequence of cysteine-rich PDZ-binding protein (CRIPT) after removal of the α3 helix [9]. A recent nuclear magnetic resonance (NMR) and small-angle X-ray scattering study of the tandem PDZ3-SH3 in PSD-95 demonstrated that the PDZ3 domain favours an orientation where its ligand binding site faces the SH3 domain, although this inter-domain interaction appears to be weak [10]. In this context, the α3 linking element can be positioned between the PDZ3 and SH3 domains or away from both of them when a C-terminal partner of PDZ3 displaces their weak interaction. Phospho-mimicking mutations at residues Tyr397 (located in the α3 helix), Ser415, and Ser418 of PSD-95 also weaken the interaction between the PDZ3 and SH3 domains [11]. PSD-95 can be phosphorylated at these sites in mouse brain, but very little is known about the consequences of these modifications *in vivo*. Considering that phosphorylation is the most common post-translational modification of proteins, the accumulation of phosphorylation sites in PSD-95 suggests that the α3-helix region is a key target for MAGUK regulation *in vivo* [11].

We demonstrated previously that residue Asp332 at the β2–β3 loop of the PDZ3 domain in PSD-95 (Figure 2) can undergo post-translational cyclation of its side chain after nucleophilic attack [12]. Succinimide ring formation, despite occurs spontaneously *in vivo* as an intermediate step in Asp isomerisation and Asn deamidation [13], likely has a functional role. For example, in the case of Asn, succinimide formation leads to spontaneous mutation to Asp [14]; also, γ-S crystallin deposits found in ocular cataracts are a consequence of reduced solubility of the protein caused by the succinilation of its Asn residues [15]. Accordingly, it has been proposed that spontaneous Asp to succinimide to iso-Asp transformations are associated with a higher β-aggregation tendency of some peptides [16], including the Alzheimer’s disease Aβ peptide [17]. The biological relevance of these post-translational modifications is underlined by the ubiquitous presence of D-aspartyl/L-isoaspartyl methyltransferase activity that renovates the Asp residues from succinimides and iso-Asp, thus avoiding possible negative consequences [18]. The half-life of succinimide rings is in the order of 1 hour, although it is affected by pH, ionic strength, electrophiles, and solvent polarity and viscosity [14]. This high degree of stability may be sufficient to influence protein activity and/or ligand binding. In the case of the PSD-95-PDZ3 domain, the succinimide ring reduces the flexibility of the β2–β3 loop and results in a loss of negative charge from the Asp332 side chain. These findings suggest that such post-translational modifications are candidate regulators of the binding affinity of the PDZ3 domain in PSD-95.

Here, isothermal titration calorimetry (ITC), NMR and molecular dynamics (MD) simulations were used to study the interaction between the PDZ3 domain in PSD-95 and the consensus hexapeptide KKETAV, which is the highest affinity binding partner of PSD-95 identified to date [19]. In addition, we have explored by site-directed mutagenesis, using experimental and computational approaches, the energetic and dynamic impact of phosphorylation at Tyr397 (P^397^-PDZ3) and succinimidation of Asp332 (SNN^332^-PDZ3) on the binding abilities of the PDZ3 domain of PSD-95.

## Materials and Methods

### Protein Samples

The plasmids encoding the PDZ3 mutants (Table 1) were derived from a wild-type PDZ3 plasmid (including residues 302–403 of the full PSD-95 protein) [20] using the QuikChange Site-Directed Mutagenesis Kit (Agilent). The 10ct-PDZ3 construct (residues 302–393 of the full PSD-95 protein) was obtained by PCR amplification, as described previously [21]. All proteins were overexpressed in *Escherichia coli* BL21/DE3 and purified as described previously [20], [22]. The KKETAV peptide used for ITC was purchased from Peptide 2.0 (USA). For NMR analyses, the samples were prepared by dissolving lyophilised protein, which was previously dialysed against water, in potassium phosphate buffer (pH 7.5) and then readjusting the pH. For all other analyses, the samples were prepared by extensive dialysis against a large volume of the same buffer. Protein concentrations were determined by measuring UV absorbance at 278 nm, using an extinction coefficient of 1492 cm^−1^·M^−1^ (Y397E-PDZ3 only) or 2985 cm^−1^·M^−1^ (all other proteins). These extinction coefficients were estimated as described previously [23]. The molecular weights and purities of the proteins were confirmed by MALDI-TOF performed by the CIC services of the University of Granada.

**Table 1:** Thermodynamic parameters of the interaction between PSD-95-PDZ3 and various ligands determined by ITC^a^.

	n	K_d_ (µM)	ΔH (kcal·mol^−1^)	−TΔS (kcal·mol^−1^)	ΔG (kcal·mol^−1^)
**PDZ3/KKETAV** b	0.99	1.5	−9.8	1.8	−8.0
**PDZ3/KKETAV** c	1.00	0.45	−5.3	−3.4	−8.7
**PDZ3/YKQTSV CRIPT** d	0.80	1.0	−10.0	1.9	−8.1
**Δ7ct -PDZ3/YKQTSV CRIPT** d	0.90	6.0	−3.4	−3.7	−7.1
**Δ10ct-PDZ3/KKETAV** b	0.90	3.6	−8.8	1.4	−7.4
**E334Q-PDZ3/KKETAV** b	1.09	2.9	−9.8	2.2	−7.6
**E401R-PDZ3/KKETAV** b	1.10	1.5	−9.2	1.2	−8.0
**Y397E-PDZ3/KKETAV** b	0.98	2.7	−7.4	−0.2	−7.6
**PDZ3/NYKQTSV CRIPT** e	0.98	3.6	−8.6	1.2	−7.4
**P^397^-PDZ3/NYKQTSV CRIPT** e	0.99	14.0	−8.8	2.2	−6.6
**D332P-PDZ3/KKETAV** b	1.05	16.0	−7.9	1.4	−6.5
**D332G-PDZ3/KKETAV** b	1.01	2.2	−8.7	1.0	−7.7

a Examples of the ITC experiments are shown in Figures 3 and S1. The variability in the experimental values was estimated to be approximately 1% for the number of binding sites, 5% for the binding enthalpy, and 10% for the binding affinity [29].

b The experimental conditions were 50 mM potassium phosphate (pH 7.5) at 25°C.

c The experimental conditions were 20 mM MES (pH 6.0) and 10 mM NaCl, at 25°C [19].

d The experimental conditions were 50 mM potassium phosphate (pH 7.5) at 25°C [28].

e The experimental conditions were 20 mM sodium phosphate (pH 6.8), 50 mM NaCl, and 1 mM EDTA, at 25°C [11].

### Isothermal Titration Calorimetry

Calorimetric titrations of the wild-type and mutant PDZ3 with the KKETAV peptide were performed at 25°C on an ITC-200 titration microcalorimeter (Microcal Inc.). The protein concentrations in the cell were either 70 µM (for wild-type PDZ3, E401R-PDZ3, E334Q-PDZ3, D332G-PDZ3, Y397E-PDZ3, and Δ10ct-PDZ3) or 200 µM (for D332P-PDZ3). The KKETAV ligand solution was prepared at 3 mM (for D332P-PDZ3) or 900 µM (for wild-type PDZ3 and the remainder of the mutants) by dissolving the lyophilised peptide in the dialysis buffer and then readjusting the pH. Titrations were made by a series of injections of 2 µl of ligand solution (Figure S1). As a reference, an independent experiment in which the calorimeter cell contained buffer only was performed with the same ligand solution to determine the corresponding heat dilution. The dilution isotherm was subtracted from that obtained for the protein titration. The binding stoichiometry (n), the dissociation constant (K_d_), and the corresponding enthalpy change (ΔH) were determined by fitting the resulting titration isotherm to a single set of identical sites binding model using ORIGIN software (OriginLab).

### Nuclear Magnetic Resonance

The ^1^H-^15^N correlation spectra were carried out at 25°C on a Varian NMR Direct-Drive System 600 MHz spectrometer (^1^H frequency of 600.25 MHz) equipped with a cryoprobe. The protein concentration was 100 µM and the results were recorded in 90% H_2_O/10% D_2_O containing 50 mM potassium phosphate buffer (pH 7.5). For the free and bounds forms of PDZ3 and Δ10ct-PDZ3, backbones were assigned using standard triple-resonance methodologies. All NMR experiments were processed using NMRPipe software [24] and analysed using SPARKY software [25]. The ^15^N and ^1^H chemical shift perturbations (Δδ_HN_) of each amide were calculated using Equation 1.
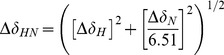
(1)


### Model Building and Molecular Dynamics

The X-ray structure of the PDZ3 domain bound to CRIPT (Protein Data Bank ID: 1BE9) was used as a template to build the model structures. To produce the coordinate files of the different experimental constructs, the C-terminal segment of PDZ3 (residues 404–415) was removed and the missing side chains were rebuilt using the design tools in Discovery Studio 2.1 Suite (Accelrys Software Inc.). To model the KKETAV ligand, the CRIPT sequence was modified as follows: the N-terminal residue was replaced by a Lys residue, Gln-3 was replaced by Glu, Ser-1 was replaced by Ala and a Val residue was added to the C-terminus. Numbering of the KKETAV peptide residues was from 0 (C-terminal Val residue) to −5 (N-terminal Lys residue). Finally, the structure was subjected to energy minimisation by the steepest descent algorithm implemented in the CHARMM simulation and analysis package. To obtain the structures of the D332P-PDZ3, Y397E-PDZ3, and P^397^-PDZ3 proteins, the side chains of the target mutated residues were modified using Discovery Studio 2.1 Suite and all models were subjected to energy minimisation using the same algorithm as that used for PDZ3.

To produce 400 ns MD simulations for the different structures, NAMD software (version 2.7b1) [26] was selected using the CHARMM22 force field. The starting structures were solvated in a 60×60×60 Å^3^ truncated octahedron box of water with the CHARMM scientific package [27]. Once solvation was complete, the structure was neutralised by replacing a number of water molecules with counter-ions to achieve the desired concentration of ions (150 mM solution of potassium chloride). The system was then minimised with a harmonic restraint at the protein atoms and crystallographic water molecules for 2000 steps, the latter to remove bad contacts of water molecules. Finally, the full system was equilibrated by carrying out conventional MD simulations with gradually weaker constraints every 1 ns on the structure (up to 4 ns).

All trajectories were generated at a constant temperature (298 K) and pressure (1 atm), under periodic-boundary conditions. Electrostatic interactions were computed using the Particle Mesh-Ewald algorithm with a real-space cut-off distance of 12 Å; this cut-off was used for the van der Waals interactions. To control temperature and pressure, the Langevin scheme was used and MD trajectories were recorded every 2 ps. The simulations were performed on the University of Granada’s UGRGrid supercomputer (a Sun Fire X2200/X4600 cluster) using 64 CPUs for each trajectory. The figures were prepared using VMD software (University of Illinois, USA).

For salt-bridge definitions, ionic interactions were considered to occur when the two last carbons before the charged atoms were closer than 5 Å. We confirmed that this approach generates equivalent results to those generated considering a 4 Å cut-off distance of the charged atoms mass centers.

## Results and Discussion

### Electrostatic Interactions between N-terminal Residues in the Ligand and the β2–β3 Loop of PSD-95-PDZ3 are Essential for Binding

To gain insight into the molecular determinants of the interactions between the PDZ3 domain of PSD-95 and its ligands, detailed thermodynamic and dynamic characterisations of the complex between the hexapeptide KKETAV and PSD-95-PDZ3 were performed. The KKETAV ligand was designed and optimised by Spaller and colleagues [19]. To our knowledge, it is the highest affinity binding partner of PSD-95-PDZ3 described to date, which demonstrates that a six-residue peptide is sufficient to capture maximal binding affinity of the PSD-95-PDZ3 domain [19], [28]. Interactions outside of the binding pocket of PSD-95-PDZ3 (Figure 2) may also be possible sources of binding specificity, as reported for other modular domains, such as the SH3 domain of the c-Abl oncogene [29].

The binding energetics of the PDZ3/KKETAV complex was measured directly using ITC. The upper panel in Figure 3 shows an example of a calorimetric titration of KKETAV to PSD-95-PDZ3 in 50 mM potassium phosphate buffer at pH 7.5. The corresponding binding isotherm is shown in the lower panel of Figure 3, together with the best fit to a one set of sites model [29]. Under these conditions, KKETAV bound to a single site with a dissociation constant of 1.5 µM (Table 1), which is approximately three times higher than that reported previously for the same ligand at pH 6.0 [19] (Table 1), suggesting that electrostatic interactions between charged residues play an important role in binding of PSD-95-PDZ3 to KKETAV. As shown in Table 1, the higher binding affinity at pH 6.0 is related to entropic contributions, which are favourable to binding at pH 6.0 but unfavourable at pH 7.5 (−TΔΔS = 5.2 kcal·mol^−1^). This entropy change overcomes the favourable contributions to binding originated from a more negative binding enthalpy at pH 7.5 (ΔΔH = 4.5 kcal·mol^−1^), reflecting the establishment of optimised interactions at higher pH.

**Figure 3:**
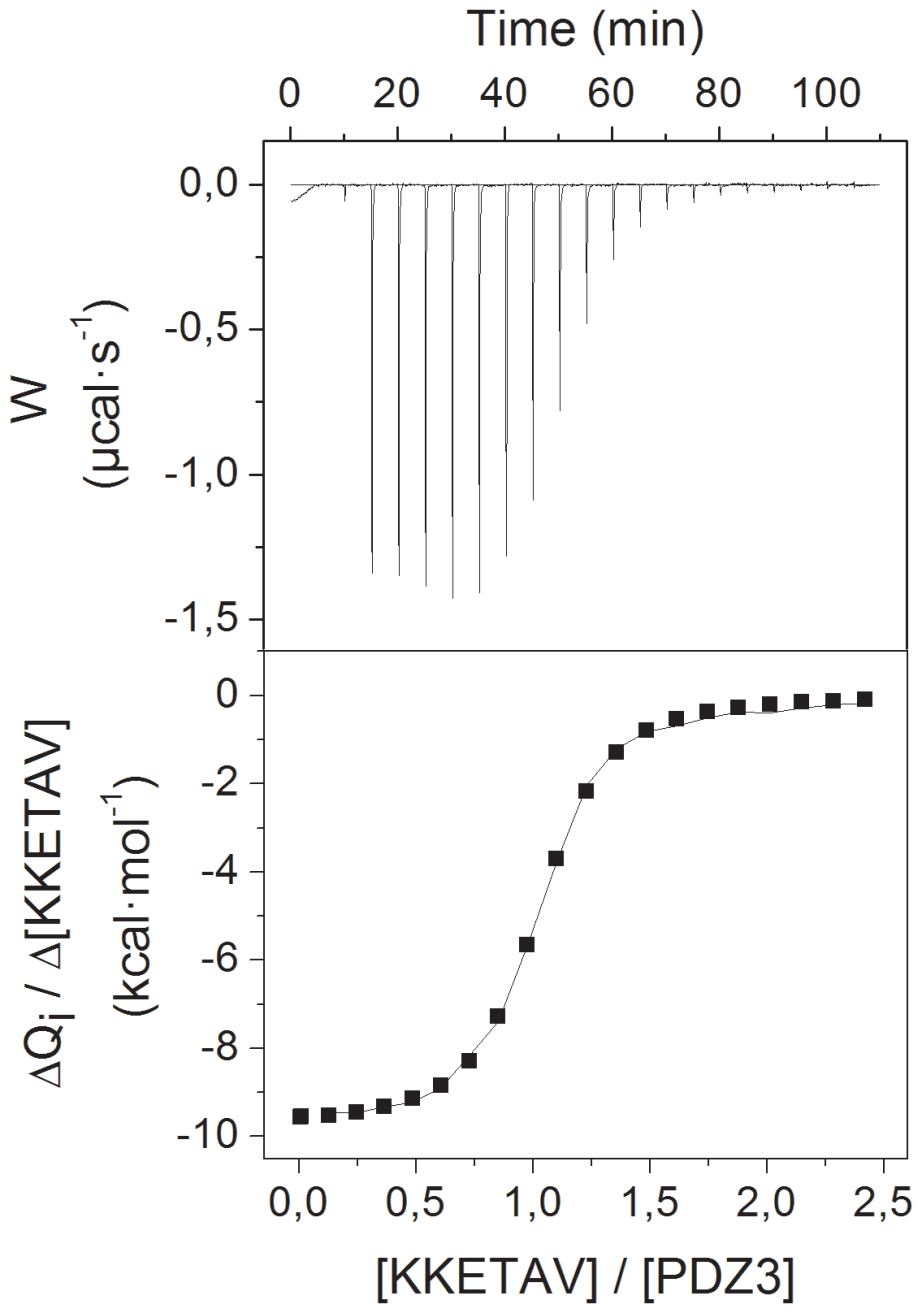
Calorimetric titration of the PSD-95-PDZ3 domain with the KKETAV ligand. Example of a calorimetric titration of KKETAV to PSD-95-PDZ3 at 25°C in 50 mM potassium phosphate (pH 7.5). The upper panel shows the net heat effects (after dilution subtraction) associated with the injection of KKETAV. The lower panel shows the ligand concentration dependence of the heat released upon binding after normalisation and correction for the heats of dilution. The trend line shows the best fit to a model considering one set of binding sites.

Analysis of a structural model generated by homology modelling of the PDZ3/KKETAV complex suggested that the pH dependency of the binding affinity is associated with the ionisation state of His372 (pKa ∼6), which is located in the α2 helix and is implicated in the formation of a highly optimised hydrogen bond with the side chain of the Thr residue in KKETAV (Figure 2). This interaction also occurs during binding of the CRIPT peptide to PSD-95-PDZ3 [30] and is considered a key interaction in class I PDZ domains [31]; however, to our knowledge, the energetic consequences of pH changes on this interaction have not yet been evaluated. Considering the lack of other titratable residues within this pH range in the vicinity of the PSD-95-PDZ3 binding site, and the fact that no substantial differences in stability and folding behaviour have been observed for the domain between pH 4.0 and pH 7.5 [32], it is reasonable to argue that entropic differences might arise from changes in the conformational entropy of ligand side chains interacting with His372 and not from significant conformational changes in the PDZ3 domain.

**Figure 4:**
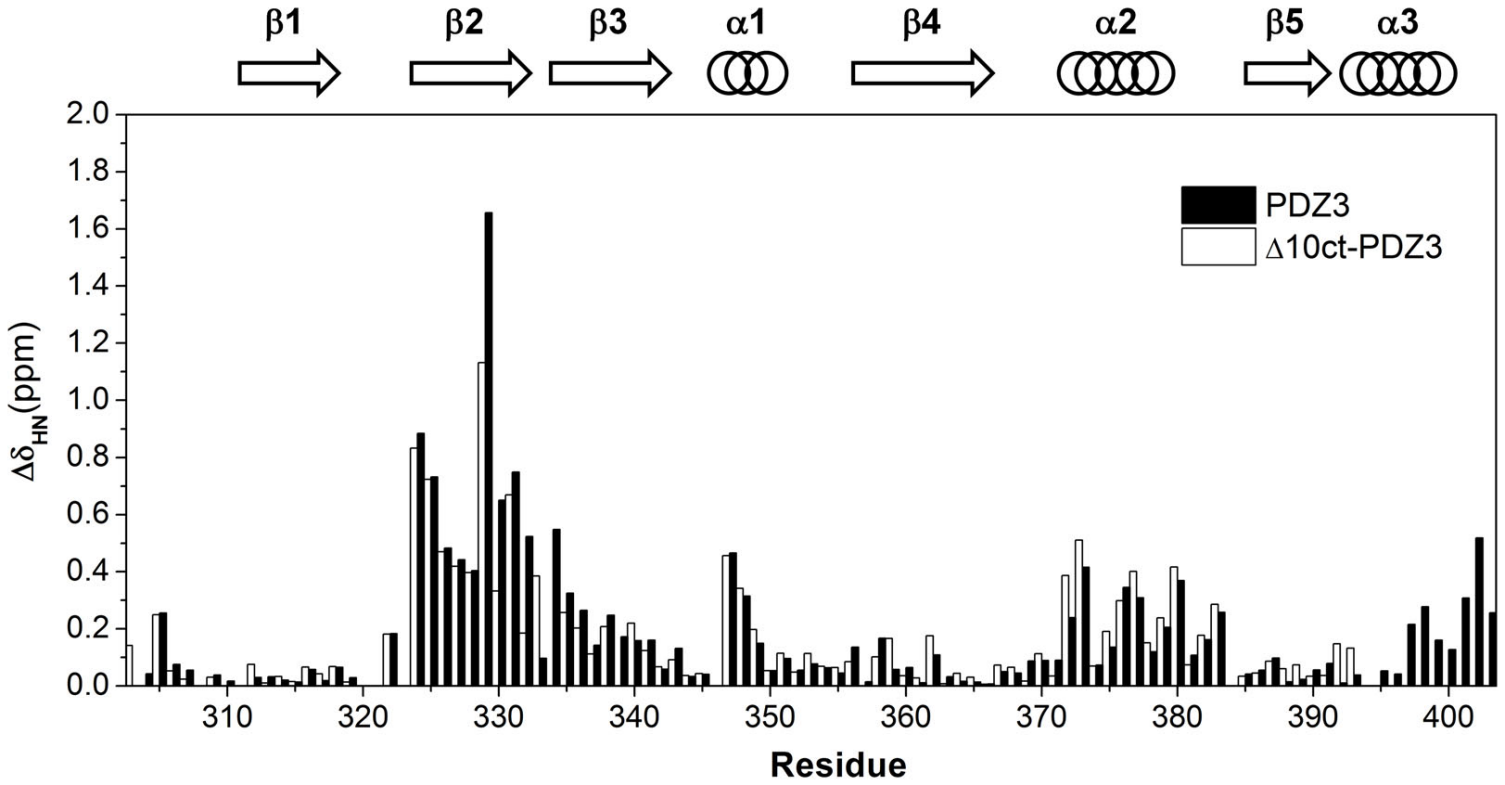
Chemical shift perturbation analyses of PSD-95-PDZ3 and Δ10ct-PDZ3 upon titration with KKETAV at pH 7.5. The ^15^N and ^1^H chemical shift perturbations (Δδ_HN_) of each amide were calculated using Equation 1 (see the Materials and Methods section). The numbers indicate the residues in PSD-95-PDZ relative to their positions in the full PSD-95 protein. The secondary structures of PSD-95-PDZ3 are shown above the graph.

Next, an NMR titration analysis was performed to determine which residues in PSD-95-PDZ3 are required for the recognition of KKETAV. It is possible to determine which residues participate in the interaction by monitoring the changes in their respective chemical shifts, because the chemical shift of every residue is related to the chemical environment surrounding the respective amide group. A series of ^1^H-^15^N heteronuclear single quantum coherence (HSQC) spectra were collected at different concentrations of KKETAV. As expected, marked chemical environment perturbations were identified for residues located in the vicinity of the binding groove of PSD-95-PDZ3 (Figures 4 and S2). However, the strongest effects were observed for residues 329–334, which are located in the β2–β3 loop. Interactions between residues in this loop of PSD-95-PDZ3 and the side chain of Lys-4 in KKETAV have been proposed previously [33]. Furthermore, binding studies have revealed a determinant role of Lys-4 in the binding affinity of KKETAV, although this interaction was not observed in the crystal structure of the complex due to lack of resolution of the Lys-4 side chain [19]. Consequently, to our knowledge, the results presented here are the first experimental proof that Lys-4 in KKETAV is essential for binding, most likely due to its interaction with the Glu331, Asp332, and Glu334 residues in PSD-95-PDZ3.

**Figure 5:**
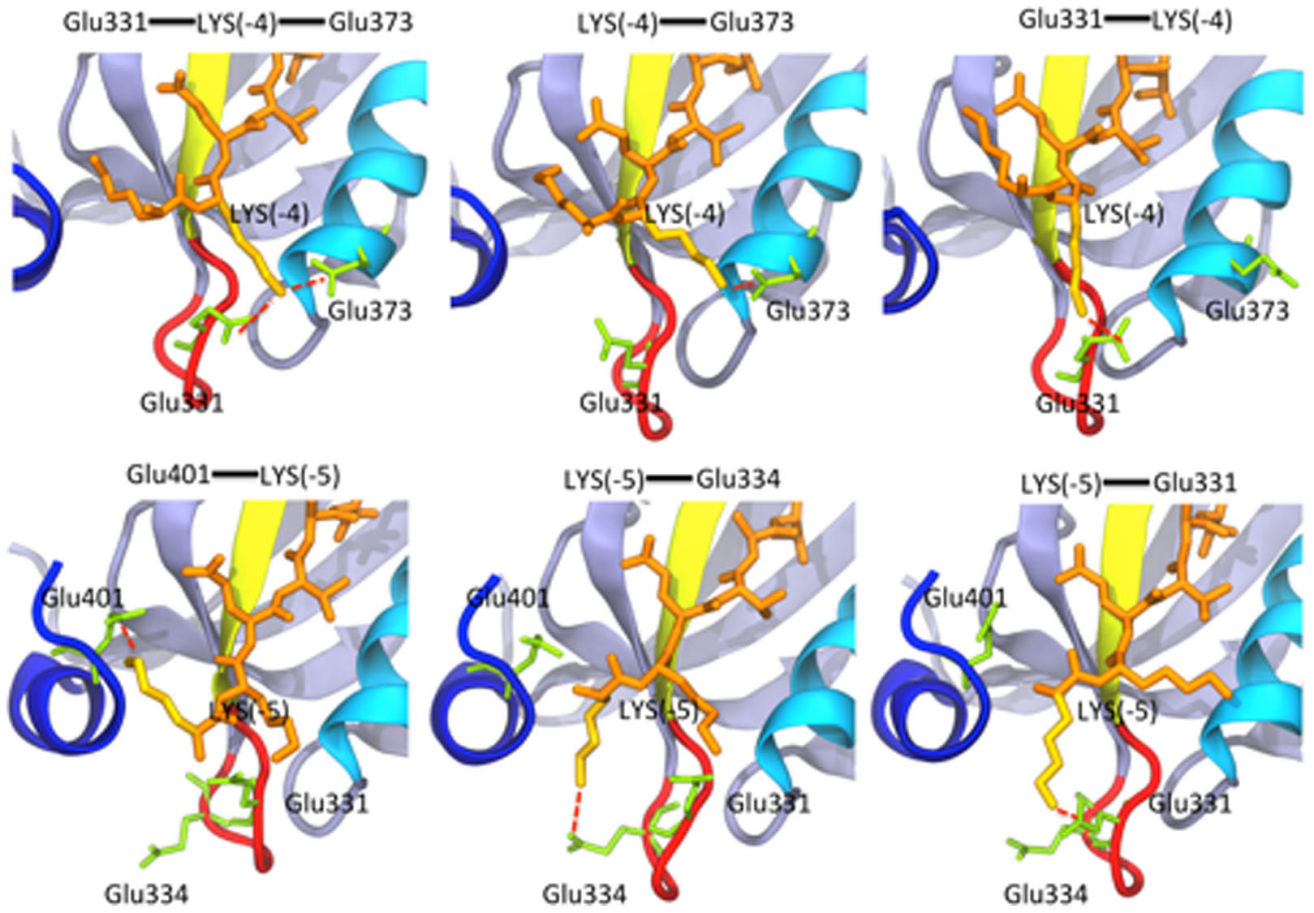
Different moments of the MD simulation of the PSD-95-PDZ3/KKETAV complex at pH 7.5. The upper panels show models of the interactions of Lys-4 in KKETAV (orange) with Glu331 and Glu373 in PSD-95-PDZ3. The lower panels show models of the interactions between Lys-5 in KKETAV and Glu331, Glu334, and Glu401 in PSD-95-PDZ3.

The interactions between residues at the N-terminus of KKETAV and the PDZ3 domain of PSD-95 were analysed further by performing MD simulations of the PDZ3/KKETAV complex. The results of this analysis are shown in Figure 5 and Table 2. Analysis of the 400 ns trajectory revealed that Lys-4 in KKETAV is involved in charge-charge interactions with Glu331 in the β2–β3 loop (observed for 37% of the simulation time) and Glu373 in the α2 helix (observed for 20% of the simulation time) of PSD-95-PDZ3. Glu331 and Glu334 in the β2–β3 loop of PSD-95-PDZ3 also interacted frequently with Lys-5 in KKETAV (20% and 36% of the simulation time, respectively), suggesting a relevant contribution of this Lys residue to the interaction. This observation is in agreement with those of previous ITC studies, which showed that adding an N-terminal Lys residue to the KETEV sequence increases the binding affinity for the PDZ3 domain of PSD-95 by an order of magnitude [34]. As shown in Table 1, elimination of the charge at position 334 of PSD-95-PDZ3 by introduction of the E334Q mutation caused a two-fold reduction in its binding affinity for KKETAV that was of entropic origin, which is typical of electrostatic interactions [35]. Globally, through this collection of salt-bridges, the interaction between the β2–β3 loop of the PSD-95-PDZ3 domain and the N-terminal region of the peptide ligand was maintained for more than 75% of the simulation time (Table 2).

**Table 2:** Frequencies of formation of some relevant salt-bridges after 400 ns of MD simulations of the PSD-95-PDZ3 constructs in complex with KKETAV.

	WT-PDZ3	Δ10ct-PDZ3	Y397E-PDZ3	P^397^-PDZ3	D332P-PDZ3
**LIGAND - β2–β3 LOOP**					
**Lys(**−**5)-Glu331**	20.0%	35.9%	8.8%	9.5%	6.1%
**Lys(**−**5)-Glu334**	36.2%	39.1%	27.2%	0.1%	0.0%
**Lys(**−**4)-Glu331**	37.2%	28.1%	51.7%	27.4%	46.3%
**LIGAND – α3 HELIX**					
**Lys(**−**5)-Glu401**	1.9%	0.0%	3.5%	5.4%	5.0%
**Glu(**−**3)-Lys403**	0.1%	0.0%	4.4%	7.7%	0.0%
**LIGAND - OTHER DOMAIN RESIDUES**					
**Lys(**−**4)-Glu373**	19.8%	16.2%	14.5%	18.6%	14.0%
**α3 HELIX - β2–β3 LOOP**					
**Lys403-Glu331**	2.7%	–	2.3%	1.8%	0.0%
**Lys403-Asp332**	0.7%	–	2.0%	0.2%	0.0%
**Lys403-Glu334**	0.0%	–	0.3%	0.0%	0.0%
**α3 HELIX - DOMAIN RESIDUES**					
**Glu334-Arg399**	39.7%	–	34.0%	44.4%	66.2%
**Lys355-Glu401**	44.3%	–	43.6%	0.0%	37.8%
**TOTAL FREQUENCY OF INTERACTIONS BETWEEN PDZ3 AND Lys(**−**4) AND Lys(**−**5) IN THE LIGAND**					
**PDZ3-Lys(**−**4)(**−**5)**	76.3%	71.8%	76.8%	54.5%	63.0%
**FREQUENCY OF NON-FORMATION OF THE Glu334-Asp399 AND Lys355-Glu401 INTERACTIONS**					
	24.9%	–	36.1%	55.6%	21.0%

The α3 extra-helix regulates ligand binding through interactions with the β2–β3 loop region. In addition to residues at the binding site and β2–β3 loop regions of PSD-95-PDZ3, NMR titrations of PSD-95-PDZ3 with KKETAV also revealed chemical shift perturbations for some residues located in the α3 helix (Figures 4 and S2), which comprises the ten C-terminal residues of PDZ3 (Figure 2). This C-terminal helix, which contains the Tyr397 phosphorylation site, is a distinctive element of the PSD-95-PDZ3 domain, as well as a few other members of the MAGUK family, not displaying the canonical PDZ fold that only contains two α-helices [30]. In principle, the binding behaviour of PSD-95-PDZ3 is not expected to be influenced by residues in the α3 helix because this helix is located at a different surface of the protein, more than 5 Å away from the binding site for residue −3 in KKETAV (looking at the 3D structure, position −3 is the closest to the α3 helix residues; Figure 2), and the protein is folded properly in its absence [21], [36]. However, deletion of this helix drastically reduces the binding affinity of PSD-95-PDZ3 for the 9-mer CRIPT peptide TKNYKQTSV [9], suggesting that it acts as an extension of the binding site for long peptide ligands containing residues at positions −5 and beyond [28]. Accordingly, a more modest effect of deletion of the α3 helix was also observed for shorter ligands [28]. Based on the results of additional NMR relaxation experiments and MD simulations, it was postulated that this reduction in the binding affinity of PSD-95-PDZ3 caused by deletion of the α3 helix is due to an allosteric effect that results in an increased conformational entropy of the PDZ3 domain [9], [33]. Here, truncation of the α3 helix of PSD-95-PDZ3 (to form the Δ10ct-PDZ3 mutant) caused a two-fold reduction in its binding affinity for the KKTEAV ligand (Table 1), which is similar to a previous report of a six-fold reduction in the binding affinity of PSD-95-PDZ3 for a shorter CRIPT fragment (YKQTSV) upon deletion of this helix [28].

Despite this inhibitory effect of deletion of the α3 helix of PSD-95-PDZ3, NMR titration experiments using ^15^N-labelled Δ10ct-PDZ3 and KKTEAV produced a chemical shift perturbation pattern very similar to that obtained for the full-length PSD-95-PDZ3 domain titrated with KKETAV (Figures 4 and S2) or the 9-mer CRIPT peptide [9]. Since the chemical environment of the PSD-95-PDZ3 residues is affected in the same way by binding of both of these ligands, a conserved binding mode that is independent of the peptide ligand and the presence of the α3 helix is plausible. As discussed in the previous section, MD simulations of the PDZ3/KKETAV complex structure did not reveal any notable interactions between the peptide ligand and the α3 helix region. Therefore, the perturbation of chemical shifts identified for residues in the α3 helix of PSD-95-PDZ3 is most likely not due to direct contacts that could eventually be established for longer ligands, rather, perturbation of the α3 helix may be caused by be an indirect mechanism.

The PDZ3/KKETAV model structure (Figure 2) includes salt-bridges between Glu401 in the α3 helix and Lys355 in the β4 strand, and between Arg399 in the α3 helix and Glu334 in the β2-β3 loop. As discussed above, Glu334 appears to play an important role in KKETAV binding via interactions with Lys-5 in the ligand. In a previous report, we proposed that these two salt-bridges are responsible for the pH dependency of the unfolding of the PDZ3 domain, which changes dramatically below pH 3.5 due to salt-bridge relaxation associated with the titration of solvent-exposed Glu residues [32]. The results presented here support the concept that the Glu401-Lys355 and Arg399-Glu334 salt-bridges play a critical role in binding linked to the α3 helix region. To investigate this hypothesis further, MD simulations of the PDZ3/KKETAV complex and the truncated Δ10ct-PDZ3/KKETAV complex lacking the α3 helix were performed. In the wild-type PDZ3/KKETAV complex, the Arg399-Glu334 and Glu401-Lys355 salt-bridges were formed for approximately 40% of the simulation time (Table 2). The Glu401-Lys355 salt-bridge did not affect binding dynamics, but the Arg399-Glu334 salt-bridge was established whenever the Glu334 side chain was not interacting directly with Lys-5 in the ligand (Figure S3). Upon the removal of the α3 helix, the interaction of Δ10ct-PDZ3 with Lys-5 in KKETAV was altered; specifically, the frequency of the interaction between Lys-5 and Glu331 was increased from 20% to 36% of the simulation time, as well as the respective between Lys-5 with Glu334 (around a 5%). Conversely, removal of the α3 helix reduced the frequency of the interaction of Glu331 with Lys-4 in KKETAV (by almost a 10%), although the interaction between Lys-4 and Glu373 at the binding site was maintained. Overall, these changes suggest modulation of the β2–β3 loop dynamics upon helix truncation. The general reduction in the interactions of the N-terminal Lys residues, including the net drop in Lys-4 interactions, may explain the reduction in binding affinity of PSD-95-PDZ3, which was mostly enthalpic, upon removal of the α3 helix.

To investigate the contribution of salt-bridges to the binding energetics of PDZ3 further, ITC was used to examine binding of KKETAV to the mutated PSD-95-PDZ3 proteins E334Q-PDZ3 and E401R-PDZ3. The E401R mutation did not affect binding (Table 1); however, the NMR studies did suggest that the α3 residues 401–403 influence the interaction between KKETAV and PSD-95-PDZ. Specifically, a chemical shift movement greater than 0.2 ppm occurred for these residues upon ligand binding (Figure 4). Since the mutation analysis indicated that Glu401 does not contribute energetically to the interaction between PSD-95-PDZ3 and its ligand, these movements may be caused by the interaction of Lys403 with several residues in the β2-β3 loop, which were detected in the MD simulations of the PDZ3/KKETAV interaction and would become weaker upon ligand binding. On the other hand, the E334Q mutation reduced the binding affinity of PSD-95-PDZ3 for its ligand by almost the same magnitude (approximately two-fold) as that caused by removal of the α3 helix (Table 1). Therefore, this single interaction between the β2–β3 loop and helix α3 seems to account for the energetic influence of the extra-helix upon ligand binding.

In summary, the results described above indicate that, although it does not establish a direct contact with the ligand, the additional α3 helix in PSD-95-PDZ3 plays a relevant role in ligand recognition through stabilisation of the β2–β3 loop in a binding-competent conformation. The following sections describe the central role of this regulatory mechanism in the modulation of PSD-95-PDZ3 binding properties through post-translational modifications.

### Phosphorylation of Tyr397 in the α3 Helix Regulates the Binding Affinity of PSD-95-PDZ3

Tyr397 in the α3 helix of PSD-95-PDZ3 has been identified previously as a phosphorylation site; therefore, we investigated the influence of this post-translational modification on the regulatory role of the α3 helix in PSD-95-PDZ3 interactions. First, we examined the effect of mutation of Tyr397 on the binding affinity of PSD-95-PDZ3 for KKETAV. The Y397E mutation reduced the binding affinity from 1.5 µM to 2.7 µM and caused a marked enthalpic change (ΔΔH_Y397E_ = 2.4 kcal·mol^−1^), which was compensated by entropic changes (Table 1). These results indicate that, although the effect of the Y397E mutation on the dissociation constant was fairly modest, considerable changes in the molecular interactions occurred. To explore these molecular changes further, MD simulations of the Y397E-PDZ3 mutant were performed. The introduction of an additional negative charge at position 397 by the Y397E mutation led to an approximate 10% decrease in the frequencies of occurrence of the interactions between Lys-5 in the ligand and the negatively-charged Glu331 and Glu334 residues in the β2–β3 loop, as well as an approximate 10% increase in the frequency of interaction between Glu331 with Lys-4 in the ligand (Table 2).

The structural model of the PDZ3/KKETAV shows that the interface between the α3 helix and the PDZ3 core contains a high density of negatively-charged residues, including Glu334 from the β3 strand, Glu396 and Glu401 from the α3 helix, and a number of others (Figure 2, right panel). The introduction of an additional negative charge at position 397 would strengthen the electrostatic repulsions and may result in opening of the C-terminal α3 helix, as suggested by a previous NMR study [36]. This concept is supported by our previous folding studies, which showed that PSD-95-PDZ3 unfolds as a two-state protein at pH values lower than 3.0, at which Asp and Glu residues are protonated [32]. At higher pH values, electrostatic repulsion caused by the negative charges present at the interface between the α3 helix and the PDZ3 core results in suboptimal packing of the α3 helix and a more complicated three-state unfolding process. In this previous study, α3 helix interactions were estimated to contribute up to 10 kcal·mol^−1^ to PSD-95-PDZ3 unfolding enthalpy [32].

Thus, the data described above suggest that Glu residues at the α3 helix/PDZ3 interface have opposing effects on protein conformation: the above mentioned repulsive effect and a favourable effect arising from the establishment of the Lys355-Glu401 and Glu334-Arg399 salt-bridges. To evaluate this α3 helix conformational flipping, MD simulations were used to estimate the total frequencies of occurrence of the Lys355-Glu401 and Glu334-Arg399 salt-bridges. Because these salt-bridges are located in the two regions where the helix is packed (the β4 strand and the β2–β3 loop), we assumed that the helix was not attached to the PDZ domain in the absence of salt-bridge formation. Conversely, when at least one of the salt-bridges was formed, we assumed that the helix was attached to the PDZ3 core. As shown in Table 2, the frequencies of non-formation of the salt-bridges in the KKETAV complexes between wild-type PDZ3 and the Y397E-PDZ3 mutant were 25% and 36%, respectively, indicating that the Y397E mutation promotes the open conformation of the protein complex. Therefore, the introduction of an additional negative charge at Tyr397 in PSD-95-PDZ affects conformational flipping between the docked and undocked forms of the α3 helix.

**Figure 6:**
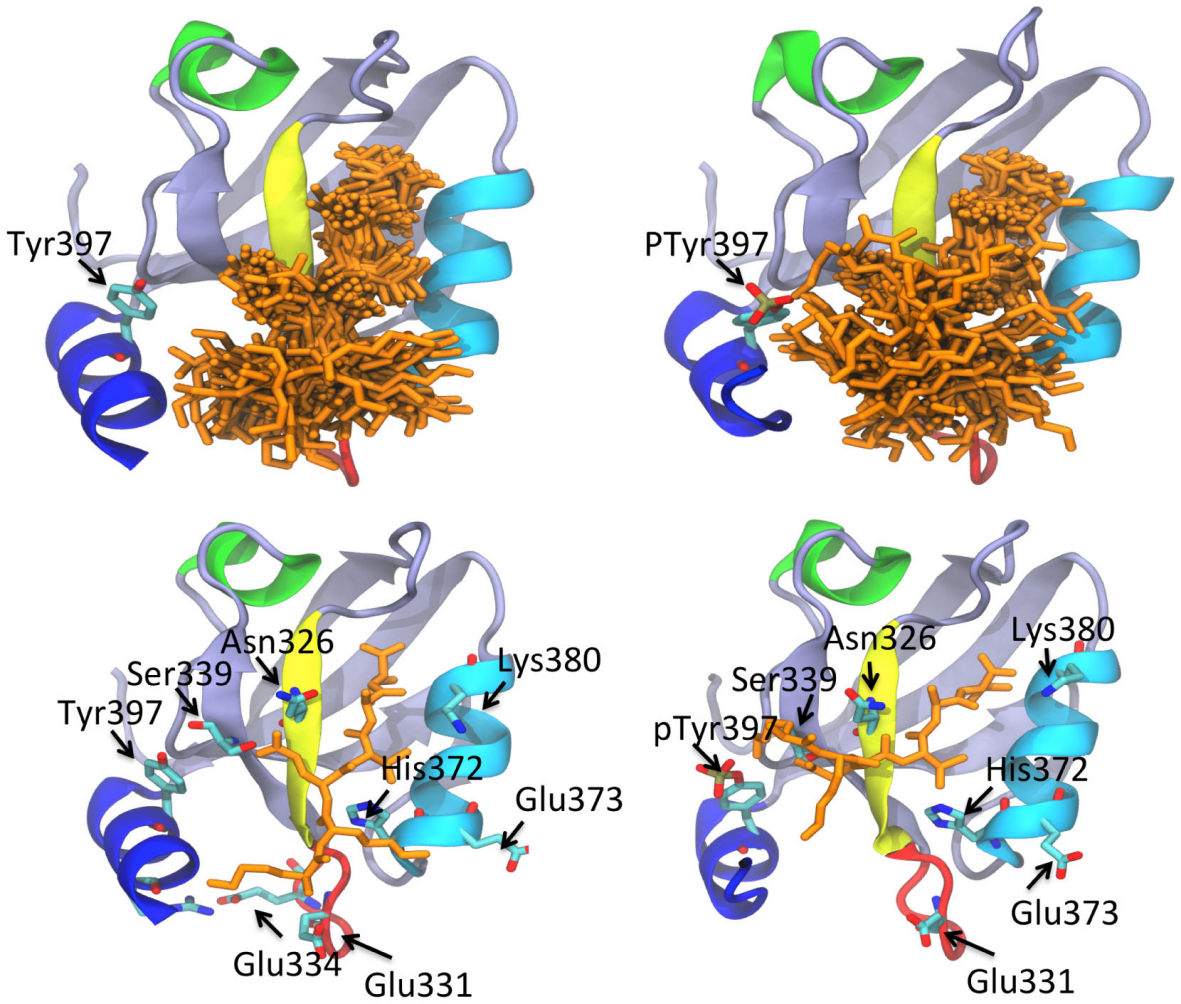
Peptide ligand freedom during MD simulations. The upper panels display peptide ligand (orange) freedom during MD simulations of the PDZ3/KKETAV (upper left panel) and P^397^-PDZ3/KKETAV (upper right panel) complexes. The lower panels display different moments of the MD simulations with the PDZ3/KKETAV (lower left panel) P^397^-PDZ3/KKETAV (lower right panel) complexes, showing the displacement of the KKETAV ligand towards phosphorylated Tyr397 residue.

Next, MD simulations of the Y397E-PDZ3 mutant and PDZ3 containing phosphorylated Tyr397 (P^397^-PDZ3) were performed. The Y397E mutation had no effect on the frequency of occurrence of interactions between Lys-4 and Lys-5 and the PSD-95-PDZ3 domain, but phosphorylation of Tyr397 caused a dramatic reduction in this frequency from 76% to 55% (Table 2). Notably, unlike the Y397E mutation, phosphorylation of Tyr397 caused movement of the ligand in the PDZ3/KKETAV complex. Specifically, the KKETAV peptide translocated from the binding pocket, such that its Lys-4 residue was positioned in close proximity to the phosphate group on Tyr397 and a salt-bridge between Lys-5 and Glu401 was formed and maintained until the end of the simulation (Figure 6). Furthermore, phosphorylation of Tyr397 caused the formation of intra-domain interactions between Lys403 at the C-terminus of the α3 helix and the negatively-charged Glu331 and Asp332 residues in the β2–β3 loop. Although we do not believe that this peptide movement reflects the real dynamic features of the interaction between PSD-95-PDZ3 and KKETAV, it does reveal the high electronegative potential generated by Tyr397 phosphorylation. This effect of Tyr397 phosphorylation was even more evident when helix flipping of PSD-95-PDZ3 was examined. The frequencies of non-formation of the Glu334-Arg399 and Lys355-Glu401 salt-bridges in the wild-type PDZ/KKETAV, Y397E/KKETAV, and P^397^-PDZ3/KKETAV complexes were 25%, 36%, and 56%, respectively (Table 2). These results indicate that phosphorylation of Tyr397 promotes helix flipping and formation of the open conformation more effectively than the Y397E mutation, most likely due to the fact that the electronegative potential is increased by the additional negative charge of the phosphate group compared to Glu. Moreover, the Y397E mutant does not contain the aromatic electrons of Tyr397 and the π-electron clouds of the aromatic rings of residues at the interface between the PDZ3 core and the α3 helix will also contribute to helix flipping to some degree. Indeed, a previous study demonstrated that phosphorylation of Tyr397 reduced the binding affinity of PSD-95-PZD3 for the CRIPT peptide from 3.6 µM to 14.0 µM [11].

Truncation of the α3 helix is not expected to replicate the reduction in binding affinity of PSD-95-PDZ3 for KKETAV caused by phosphorylation of the Tyr397 residue because helix flipping will not occur and the absence of this helix will not reproduce the almost pure entropic nature of the reduction in affinity caused by the additional phosphate group. Furthermore, another entropic source may also contribute to the increased freedom that the peptide experiences at the binding pocket upon inclusion of the extra phosphate group (Figure 6). These entropic factors were not observed upon deletion of the α3 helix. The binding affinity of Δ10ct-PDZ3 for KKETAV was only two-fold lower than that of wild-type PDZ3; this reduction was mainly enthalpic in nature (Table 2) and, as mentioned above, can be qualitatively explained by redistribution and reduction of the interaction frequencies of Lys-4 and Lys-5 in the ligand. Overall, the results presented here demonstrate that the β2–β3 loop plays a critical role in PDZ3 binding due to direct interaction with the peptide and with the α3 helix. They also show that phosphorylation can regulate binding by disturbing the helix, with the concomitant alteration of the β2-β3 loop.

### Succinimide Cyclation of the Asp332 Side Chain Affects the Binding Properties of PSD-95-PDZ3

As mentioned earlier, cyclation of the Asp332 residue to a succinimide ring may affect the binding affinity of PSD-95-PDZ3. Succinimide formation is a spontaneous chemical modification of Asp and Asn residues located preferentially at loops or exposed regions in proteins in which the side chain cyclises. We reported previously that Asp332 in the β2-β3 loop of PSD-95-PDZ3 can undergo spontaneous succinimide formation at neutral pH during the crystallisation process [12]. To determine if this succinimide ring is stable in solution, a collection of SNN^332^-PDZ3 crystals were dissolved in 50 mM potassium phosphate (pH 7.5) buffer and the spontaneous side chain linearisation kinetics were followed by mass spectrometry. The molecular weight reduction of 18 Da associated with succinimide formation was used as an indicator of the population of linearised side chains at different time points. The mass spectrometry peak corresponding to the molecular weight of succinimide was reduced by almost 50% after 1 h and disappeared fully within 24 h. These data indicate that, despite its transient nature, the succinimide ring in PSD-95-PDZ3 is stable enough to influence its binding properties. Unfortunately, the kinetic nature of the process precludes a direct experimental investigation of its effects on binding thermodynamics. Considering that the proline ring is structurally very similar to the succinimide ring, and that Asp332 does not interact with Lys residues in the peptide ligand or with the α3 helix residues in PSD-95-PDZ3, we generated and used the D332P-PDZ3 mutant as an acceptable model of succinimide formation. The effects of this mutation on the binding energetics and conformational equilibrium of the protein were examined by MD simulations.

ITC analyses of the interaction between KKETAV and D332P-PDZ3 in 50 mM potassium phosphate (pH 7.5) revealed that the D332P mutation reduced the binding affinity of PDZ3 for KKETAV dramatically (wild-type PDZ3, 1.5 µM; D332P-PDZ3, 16.0 µM), as reflected by a less favourable binding enthalpy (Table 1). In agreement with this result, MD simulations also showed that this mutation reduced the percentage of time during which Lys-4 and Lys-5 in the ligand formed at least one salt-bridge with PSD-95-PDZ3 from 76% to 63% (Table 2). Notably, the reduction in binding affinity caused by the D332P mutation was associated with almost complete loss of the salt-bridges between Lys-5 in the ligand and the Glu331 and Glu334 residues in the β2–β3 loop of PDZ3, which were established in the wild-type PDZ3/KKETAV complex for 20–36% of the simulation time (Table 2). Conversely, the D332P mutation did not affect the interaction between Lys-4 and PDZ3 or the helix flipping frequencies (Table 2). Therefore, the lower binding affinity of D332P-PDZ3 compared with wild-type PDZ3 may be due to a reduction in the conformational freedom of the β2-β3 loop caused by the proline ring, which impairs the establishment of interactions.

The results described above suggest that the β2–β3 loop of PSD-95-PDZ3 is a key determinant of binding affinity. To provide additional evidence for this theory, we determined the effect of the D332G mutation on the binding affinity of PSD-95-PDZ3 for KKETAV. The D332G mutation caused a loss of binding affinity similar to that caused by the E334Q mutation (Table 1), indicating that the additional conformational freedom in the β2–β3 loop caused by the glycine residue has similar effect on binding affinity as relaxation of the Glu334-Arg399 salt-bridge. These results support the concept that the dynamic properties of the β2–β3 loop determine the binding affinity of PDZ3 and that the α3 helix exerts a regulatory effect simply by influencing this dynamic behaviour through the Glu334-Arg399 interaction. From a dynamic point of view, succinimide formation has a similar effect on binding but is achieved in a different way, namely by limiting the conformational freedom of the β2–β3 loop. Although mutations may not be relevant to regulation of PDZ3 binding *in vivo*, similar effects may be achieved by post-translational modifications, such as those described here.

### The Effects of Post-translational Modifications on the Regulation of PDZ3 Binding

Although it is not the first example of modulation of the binding activities of PDZ family members [37]–[41], the regulation of the α3 helix of PSD-95-PDZ3 has some interesting features. As demonstrated here and in previous studies, the absence of the α3 helix does not drive conformational changes in PDZ3 [9], [21], [36]. Nevertheless, the α3 helix modulates the dynamics of the side chains, mainly those of Glu334 and Arg399 that connect this extra-helix with the β2–β3 loop. According to the MD analyses described here, the dynamics of this loop is central to the control of binding of linear peptides to PDZ3, since positively-charged residues in ligands at positions −4 or beyond interact with the negative carboxylates of Glu331 and Glu334 in the β2–β3 loop. This interaction network is essential for binding because peptides shorter than four residues do not bind to PDZ3 [19]. Previous MD simulations have shown that PDZ3/peptide binding is also regulated by the formation of an additional salt-bridge between residues Lys355 and Glu401, which are postulated to connect the α3 segment with the well-conserved carboxylate binding loop through a hypothetical network of interactions within PDZ3 [33]. However, as mentioned above, the E401R mutation did not affect the binding affinity of PSD-95-PDZ3 for the KKETAV peptide (Table 1).

The results presented here suggest that the modulatory effect of the α3 linker sequence (which connects the SH3 domain and the remainder of the PDZ3 and is positioned far away from the binding pocket) on the binding affinity of PSD-95-PZD3 is not caused by a conformational change or the existence of a intramolecular network. On the contrary, it seems that this modulatory effect is attributable to a much simpler mechanism involving a salt-bridge that connects the α3 helix to the β2–β3 loop. Moreover, the formation of this salt-bridge competes with others established between three negatively-charged residues of the β2–β3 loop and two positively-charged residues in the peptide ligands. These positive charges are well conserved amongst different peptide ligands described for PSD-95-PDZ3, all of which contain at least one Lys and/or one His residue at positions −4 and beyond [19]. This relatively simple mechanism explains the reduction in the binding affinity of PSD-95-PDZ3 for KKETAV upon phosphorylation of Tyr397, which is located in the α3 helix. As mentioned above, the interface between the α3 helix and the PDZ3 core contains a high density of negative charges (Figure 2, right panel), resulting in electrostatic repulsion under neutral pH conditions. The π-electron clouds of the aromatic rings of Phe337 in the β3 strand and Tyr397 and Phe400 in the α3 helix may also form anion-π interactions that contribute to the electrostatic repulsion (Figure 2, right panel). Therefore, as described above, the introduction of additional negative charge through phosphorylation of Tyr397 promotes flipping between the docked and undocked forms of the α3 helix. This strong interfacial negative potential is supported by substantial conformational freedom of the peptide, which is able to pull out from the binding pocket, as demonstrated by MD simulations with P^397^-PDZ3. These effects translate into a reduction in the net frequency of interactions between both Lys residues of the peptide ligand and the negatively-charged residues of the β2-β3 loop in PDZ3, thereby reducing the binding affinity in a mainly entropic manner.

The reduced binding affinity of the D332P-PZD3 mutant, which simulated cyclation of Asp332 into a succinimide ring, is comparable to the binding affinity reported for the P^397^-PDZ3/CRIPT complex in a previous study [11]. Asp332 did not interact with any residue of the KKETAV ligand, but mutating Asp332 modulated the conformational properties of the β2–β3 loop and consequently the stability of the salt-bridge network between this loop and the positively-charged residues in the ligand. The dynamics of the α3 helix did not seem to affect binding in this case, since the formation of the Lys355-Glu401 and Glu334-Asp399 salt-bridges was not affected appreciably by the D332P mutation (Table 2).

## Conclusions

In summary, interplay of the salt-bridges between the α3 helix and β2–β3 loop of PSD-95-PDZ3 and the positively-charged residues of the ligand peptide can account for the regulatory effects of post-translational modifications of PDZ3, both energetically and dynamically. The observed interactions between residues in the α3 helix and the β2–β3 loop may exist in nature when the SH3 domain that follows the α3 helix is present. Therefore, it is possible that the C-terminal tails of ligand proteins compete with the salt-bridge network. The positive charges at the C-terminus of a ligand protein will contribute to the disorganisation of the naturally-occurring inter-domain associations. In addition, the chemical shift dispersion with the KKETAV peptide described here is similar to that reported for the CRIPT peptide previously [9], suggesting that this regulatory mechanism is universal for ligand binding to PDZ3. Overall, the results presented here demonstrate that post-translational modifications of PSD-95-PDZ3 are potent and simple regulators of ligand binding that might trigger the delivery or capture of a target, thereby facilitating the complex biological role of the MAGUK protein PSD-95 *in vivo*.

## Supporting Information

Figure S1 **Calorimetric titrations of the PSD95-PDZ3 mutants with the ligand KKETAV at 25 °C in 50 mM potassium phosphate (pH 7.5).** Upper panels: net heat effects, after dilution substraction, associated with the injection of KKETAV (see Materials and Methods for details). Lower panels: ligand concentration dependence of the heat released upon binding after normalization and correction for the heats of dilution. Symbols represent experimental data and the continuous line corresponds to the best fitting to a model considering one set of binding sites.
(DOCX)

Figure S2 **Chemical shift perturbation experiments of PDZ3 and Δ10ct-PDZ3 upon titration with KKETAV at pH 7.5.** The panels show the extent of the perturbation for every residue of PDZ3 (left) and Δ10ct-PDZ3 (right) as a colour code: blue for Δδ values ranging from 0 to 0.2 ppm; yellow for 0.2 to 0.5 ppm; red for Δδ values higher than 0.5 ppm.
(DOCX)

Figure S3 **Distance values between residue Glu334 and either Lys-5 (red) or Arg399 (blue) along the 400 ns trajectories from the MD simulations performed with PDZ3/KKETAV.**
(RAR)
