# Supplementary figures and images for: Post-Translational Modifications Modulate Ligand Recognition by the Third PDZ Domain of the MAGUK Protein PSD-95
Source: PLoS One. 2014 Feb 26;9(2):e90030. doi: 10.1371/journal.pone.0090030 (PMC3935999; doi:10.1371/journal.pone.0090030)

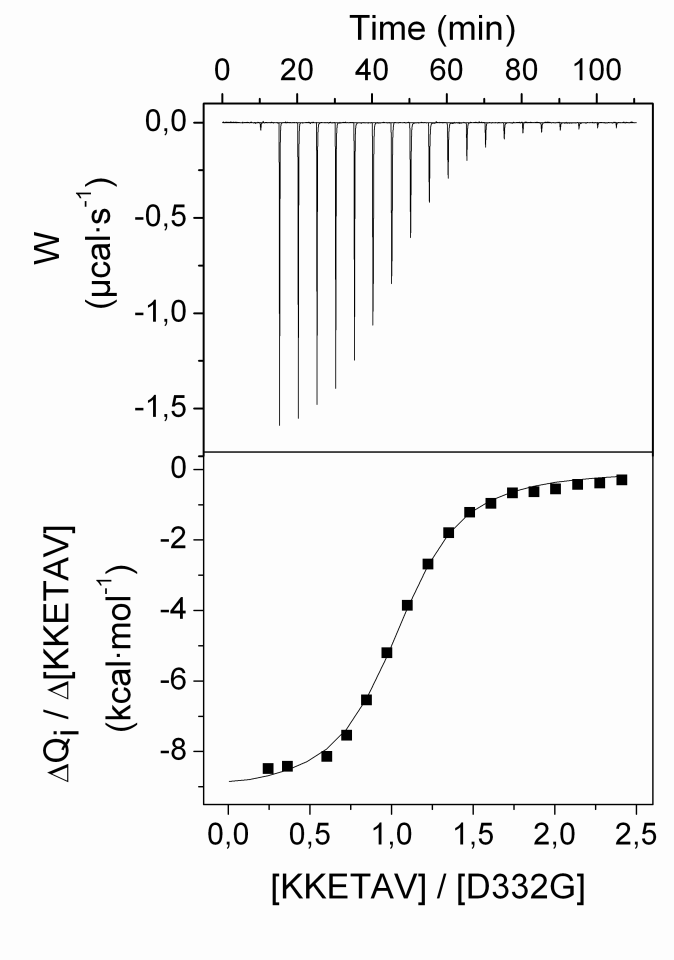

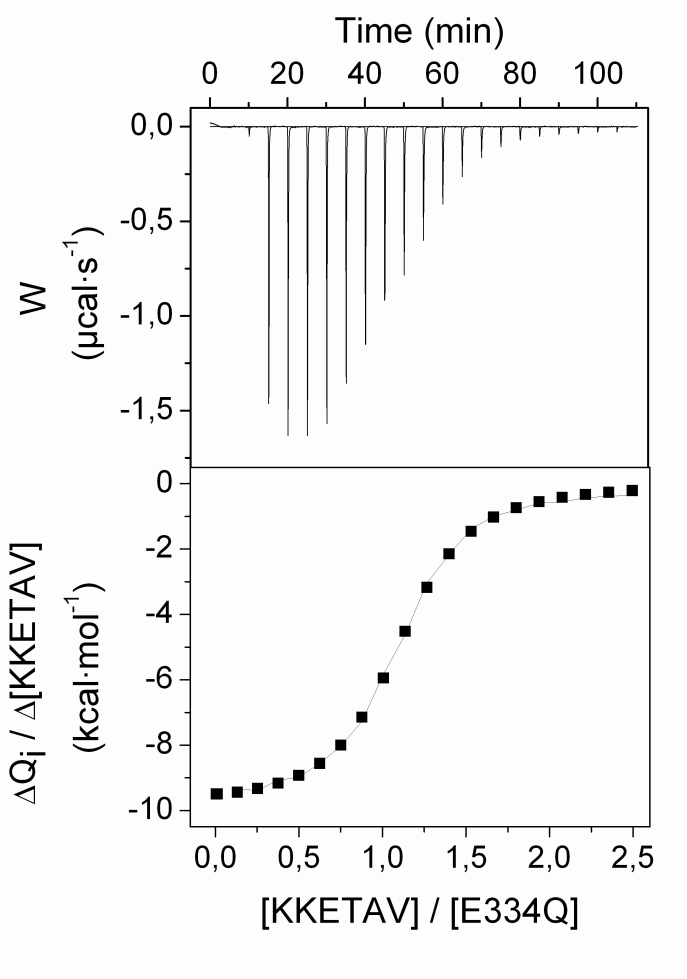

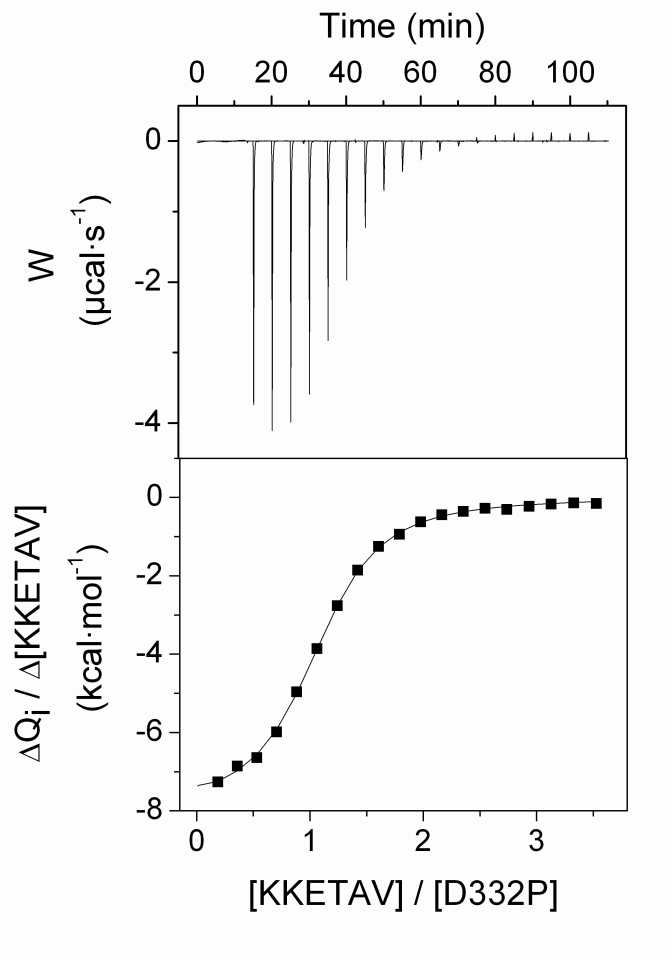


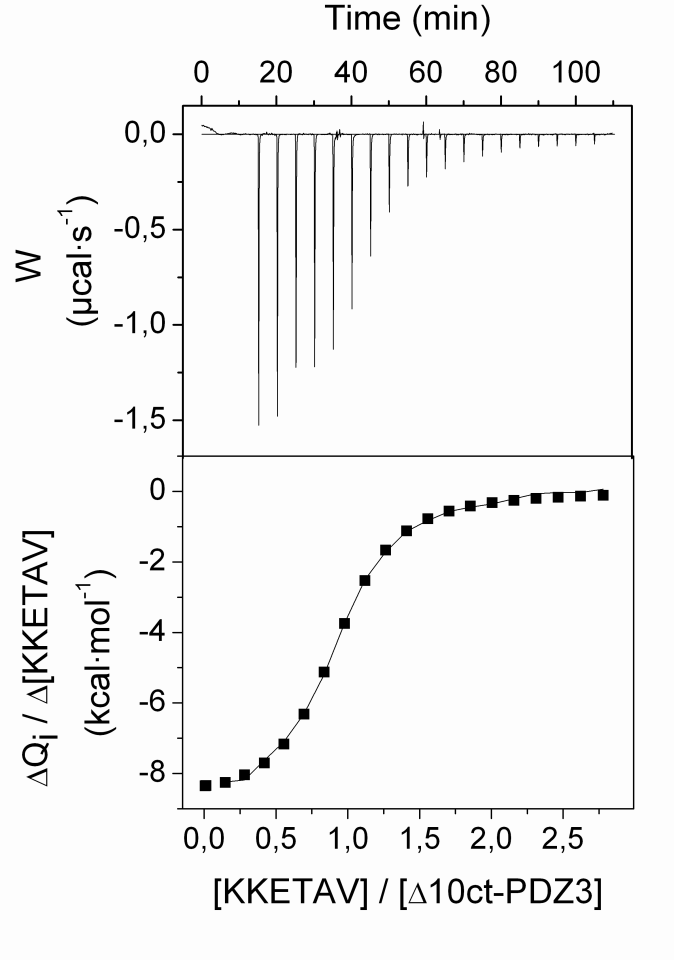

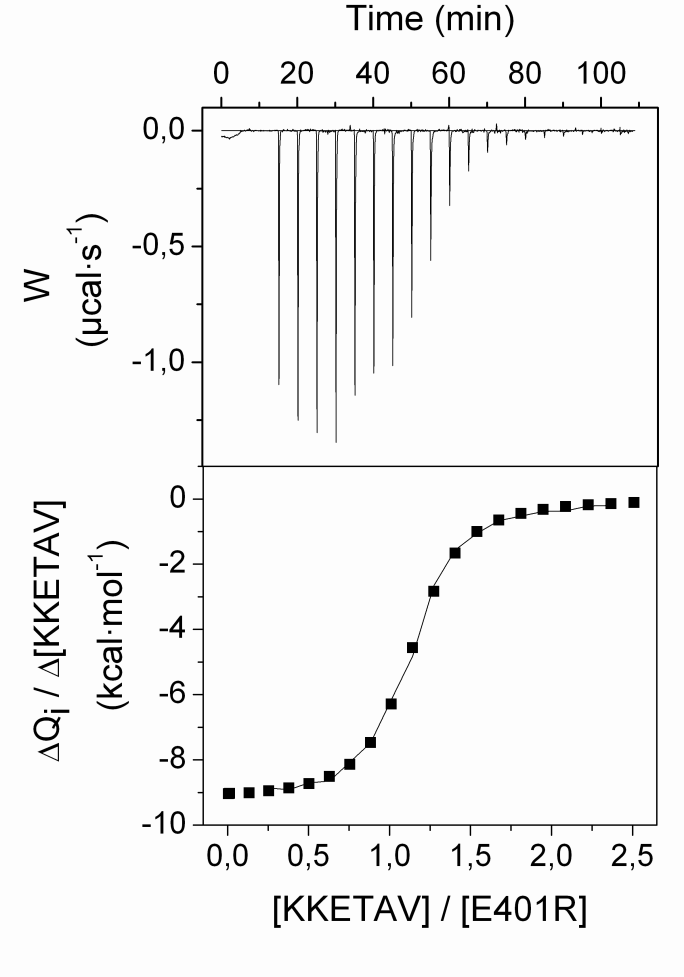

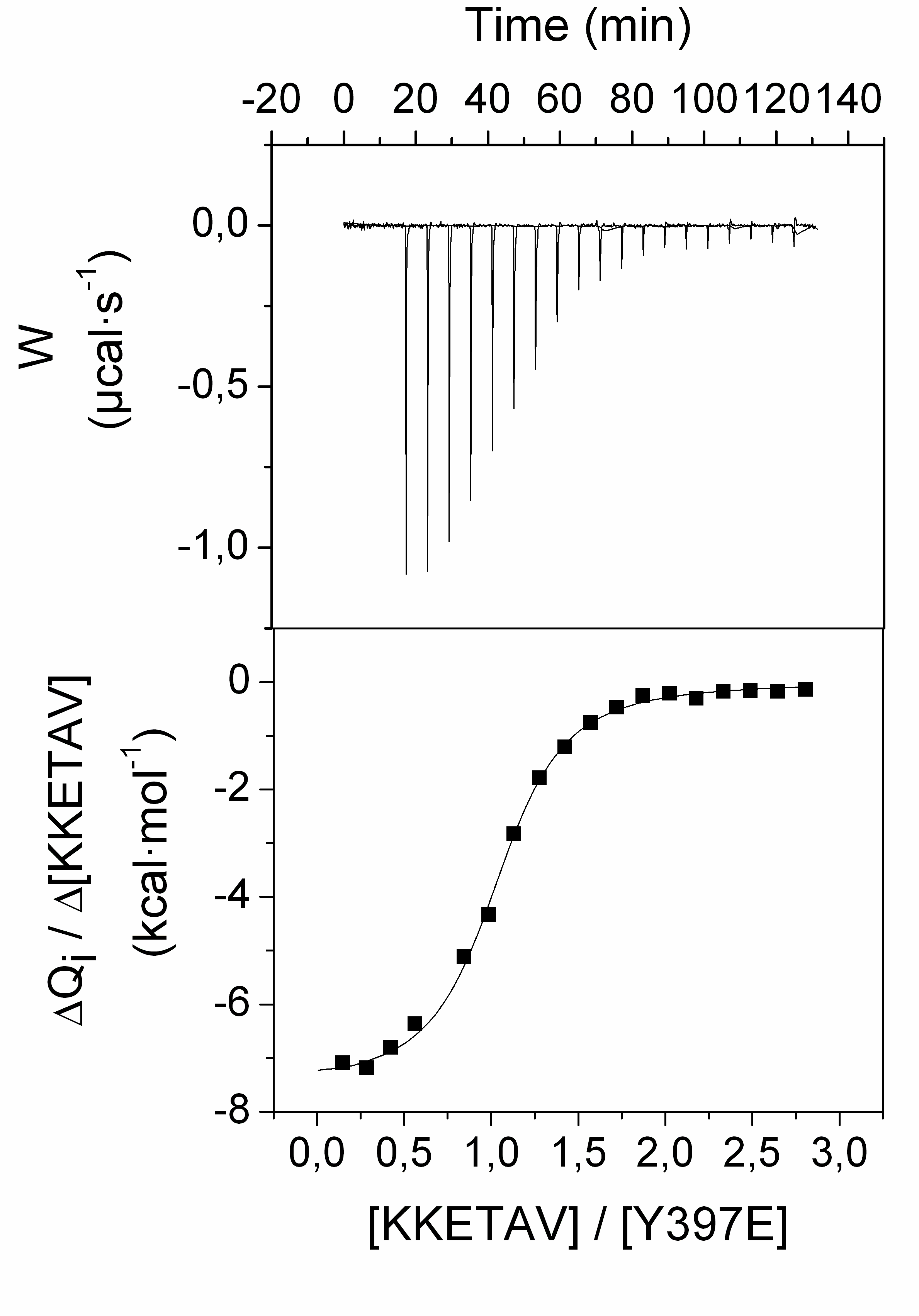

Supplement: Figure S1 — Calorimetric titrations of the PSD95-PDZ3 mutants with the ligand KKETAV at 25 °C in 50 mM potassium phosphate (pH 7.5). Upper panels: net heat effects, after dilution substraction, associated with the injection of KKETAV (see Materials and Methods for details). Lower panels: ligand concentration dependence of the heat released upon binding after normalization and correction for the heats of dilution. Symbols represent experimental data and the continuous line corresponds to the best fitting to a model considering one set of binding sites. (DOCX) [file pone.0090030.s001.docx]

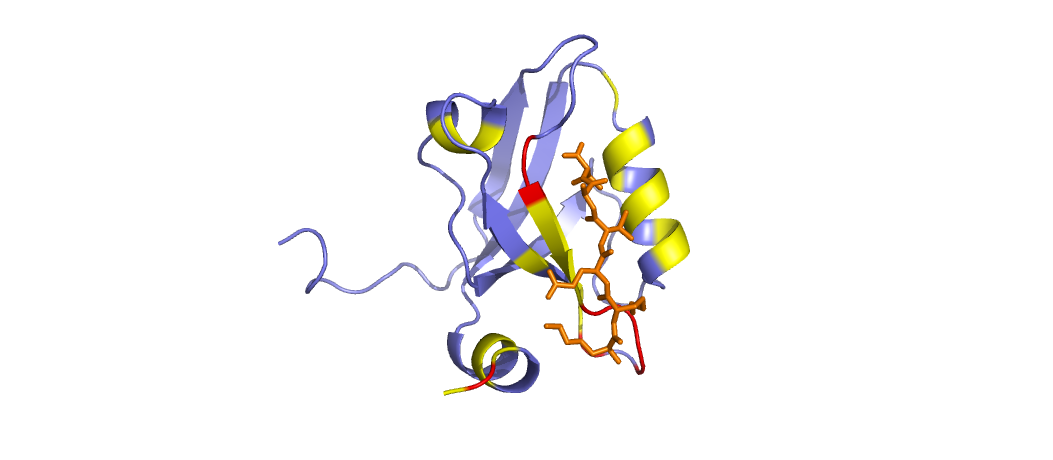

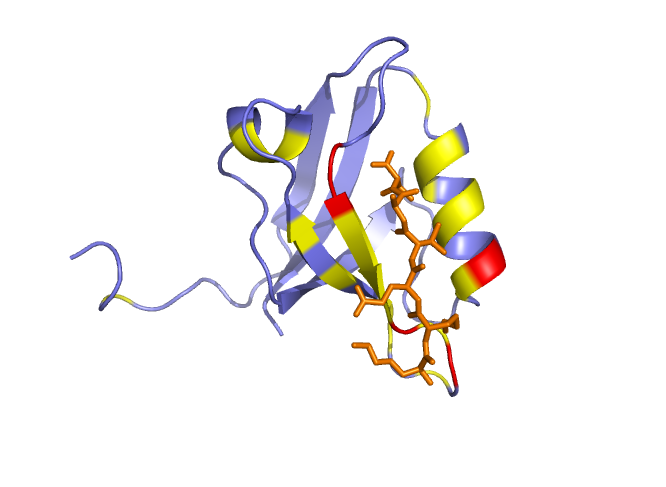

Supplement: Figure S2 — Chemical shift perturbation experiments of PDZ3 and Δ10ct-PDZ3 upon titration with KKETAV at pH 7.5. The panels show the extent of the perturbation for every residue of PDZ3 (left) and Δ10ct-PDZ3 (right) as a colour code: blue for Δδ values ranging from 0 to 0.2 ppm; yellow for 0.2 to 0.5 ppm; red for Δδ values higher than 0.5 ppm. (DOCX) [file pone.0090030.s002.docx]
